# Difference in out-of-pocket health expenditure among Syrian refugees in Jordan, Lebanon and the Kurdistan region of Iraq

**DOI:** 10.3389/frhs.2026.1812745

**Published:** 2026-06-04

**Authors:** Samaha Masroor Saqib, Rashed Mohammad Mahfuzullah, Shirin Ziaei, Soorej Jose Puthoopparambil

**Affiliations:** 1 Department of Women’s and Children’s Health, Uppsala University, Uppsala, Sweden; 2 Department of Food Studies, Nutrition and Dietetics, Uppsala University, Uppsala, Sweden

**Keywords:** health inequalities, healthcare, Jordan, Kurdistan region of Iraq (KRI), Lebanon, out-of-pocket (OOP) expenditure, Syrian refugees

## Abstract

**Background:**

Refugees frequently face financial barriers to healthcare, with out-of-pocket (OOP) payments representing a major obstacle to access. While studies often examine refugees’ healthcare access within single-country settings, less comparative evidences exist on how host-country health system structures influence refugees’ financial barriers to healthcare. Consequently, this study assesses differences in OOP healthcare expenditure among Syrian refugees residing in Jordan, Lebanon, and the Kurdistan Region of Iraq (KRI).

**Methods:**

We conducted a cross-sectional analysis using data from the “Syrian Refugee and Host Community Surveys, 2015–16”. The analytic sample included a total of 2,679 Syrian refugee households from Jordan, Lebanon and KRI, that reported at least one healthcare visit in the previous 12 months. The outcome was any reported OOP payment at the point of care and the host country being the main exposure. Generalized Estimating Equations were used to estimate crude and adjusted odds ratios, accounting for clustering and sociodemographic covariates.

**Results:**

Cross-country variation in OOP expenditure was observed., where, refugee households reporting OOP payments were 39.5% in Jordan, 78.3% in Lebanon, and 73.3% in KRI. After adjustment, refugee households in Lebanon had over five times higher odds of incurring OOP payments compared with Jordan (AOR: 5.25, 95% CI: 1.57–17.5), while those in KRI had more than four times higher odds (AOR: 4.50, 95% CI: 1.34–15.1).

**Conclusions:**

Healthcare affordability among Syrian refugees differed markedly by host-country context, highlighting important cross-country differences in financial exposure to healthcare costs. These findings underscore the relevance of host-country setting in shaping refugees’ experience of healthcare affordability and point to the need for greater financial protection to mitigate healthcare-related hardship in protracted displacement contexts.

## 1 Introduction

Forced migration refers to migratory movement which, although the drivers can be diverse, involves force, compulsion, or coercion (1). It has reached unprecedented levels, with an estimated 123.2 million individuals forcibly displaced worldwide as of 2024. Approximately one-third of this population, representing 36.9 million individuals, have crossed international borders and are classified as refugees (2). The presence of large refugee populations poses complex and multidimensional challenges that extend beyond immediate humanitarian needs, encompassing political, economic, sociocultural, and environmental domains, especially when displacements are increasingly becoming protracted (3, 4).

The health of refugees is a critical area of concern, as they are particularly vulnerable to poor health outcomes due to disrupted health systems, substandard living conditions, and limited access to healthcare services (5, 6). Displacement frequently interrupts access to essential healthcare services, such as preventive services, continuity of chronic disease management, and emergency care, while simultaneously exposing populations to deteriorating living conditions, food insecurity, and psychosocial stress (7). Seventy-three percent of all refugees are hosted by low- and middle-income countries (LMICs) (2). In many such LMIC contexts, health systems are already under-resourced and struggling to meet the needs of the local population. The sudden influx of large numbers of displaced people exacerbates these strains, both for refugees and the host population (8). In many contexts, refugees might be disproportionately affected because of additional barriers such as legal restrictions, financial hardship, and social marginalization (9, 10). This can result in refugee populations having poor access to health services (6).

One key component of healthcare accessibility is affordability, commonly measured by out-of-pocket (OOP) healthcare expenditures. OOP payments for healthcare services refer to direct payments made at the point of service by individuals or households (HHs) for any healthcare services or goods, excluding any reimbursement from insurance or third parties (11, 12). Financial constraints can lead to healthcare avoidance or delays, exacerbating health problems and threatening survival (13). Furthermore, substantial OOP spending can result in catastrophic health expenditure (CHE), where individuals or HHs are forced to sacrifice basic needs or fall into poverty to pay for care. Inability to afford OOP costs may result in delay or avoidance in seeking care, relying on informal providers, or prioritizing urgent over preventive services, thereby exacerbating health risks (14). High OOP expenditures are recognized as significant barriers to healthcare access, particularly for refugee populations whose financial resources and employment opportunities are often precarious (15, 16). Measuring OOP expenditure therefore can provide an understanding of the financial protection available to refugee HHs in the host countries. Although the absolute amounts of OOP expenditure provide useful insights, it is less comparable as it is highly context-specific, influenced by variations in service pricing, purchasing power, and reporting accuracy across countries. However, whether or not individuals make such payments is an important indicator of financial protection and equity in healthcare access, particularly in humanitarian settings where some populations benefit from subsidies, donor assistance, or camp-based services, while others have to make OOP payments for health services (14, 17).

Syrian refugees constitute one of the largest displaced groups in the world, reflecting the scale and longevity of the Syrian conflict that began in 2011 with socio-political unrest that evolved into a protracted and complex conflict marked by regional and international involvement. By the end of 2024, the number of Syrian refugees and asylum-seekers stood at 6.1 million, with nearly 80 per cent of them being hosted in neighboring countries and millions more displaced internally within Syria (2). Jordan, Lebanon, and the Kurdistan Region of Iraq (KRI) have each hosted large numbers of these Syrian refugees (2), and they highly differ in their health system structures, financing models, and humanitarian support arrangements. Jordan has a relatively institutionalized health sector, though refugees' access remains shaped by cost-sharing requirements and insurance schemes, with healthcare expenses partially paid OOP by refugees themselves (18, 19). Lebanon, on the other hand, has a highly privatized healthcare system, with limited public sector capacity and fragmented financing mechanisms that leave both host and refugee populations heavily reliant on OOP payments (75, 76). In contrast, the KRI presents a mixed model, with a central role for public provision but constrained resources, creating variable access to healthcare across different districts (20, 21).

While most Syrian refugees in Jordan reside in formal camps, those in Lebanon live within host communities. On the other hand, KRI represents a mixed approach, hosting both camp-based and non-camp refugee populations (22). Camp vs. non-camp settings add further complexity, as camp-based refugees may benefit from subsidized or free services provided by humanitarian actors operating in the camps, whereas non-camp refugees are more reliant on national systems and thus more likely to incur OOP expenditures (23, 24). These variations also add to the different levels of humanitarian support for Syrian refugees across the three countries due to differences in state capacity, integration policies, and the roles of international and non-governmental actors. Jordan has implemented a relatively coordinated response that enables partial integration of refugees into public services, supported by partnerships with UN agencies and humanitarian organizations (25, 26). In contrast, Lebanon's limited integration of refugees into national systems and constrained public sector has resulted in greater reliance on Non-Governmental Organization (NGOs) and international actors for healthcare and social services (27, 28). In the KRI, humanitarian assistance operates through a hybrid model shaped by regional governance, with services delivered through a combination of camp-based and host-community facilities, often with variable levels of funding and system integration (29, 30).

Given that Jordan, Lebanon, and KRI have different health system structures and levels of humanitarian support, with variation in camp policies, it is critical to examine how these differences affect OOP healthcare expenditures for the refugees, and what lessons can be learned from each of these three different systems. These variations underscore why comparing OOP across Jordan, Lebanon, and the KRI offers a valuable lens into how the reception context, rather than refugee characteristics alone, can influence healthcare affordability. This comparison can illuminate how healthcare access barriers, such as financial strain, vary by country and inform efforts to reduce health inequalities.

Despite the growing body of research on refugee health, most studies focus on single-country contexts (31–33), with limited comparative evidence on how different host-country health systems shape financial exposure to healthcare. The differences in host system structures provide a natural context for examining how the displaced population (Syrians), experiences healthcare access across three distinct settings (Jordan, Lebanon, and the Kurdistan Region of Iraq), despite being exposed to the same conflict in Syria. Unlike refugee studies that often involve populations displaced to a given country, but come from different contexts and are exposed to different conflicts (34, 35), our study enables to explore three different hosting country characteristics can affect healthcare affordability for refugees originating from the same country. Accordingly, the aim of the study is to assess differences in OOP healthcare expenditures among Syrian refugees residing in Jordan, Lebanon, and KRI. To address this, we used a comparative analytical approach to assess OOP payments across the three settings. By comparing these expenditures across different host country contexts, this research seeks to explore how host country context influences OOP and to contribute to the evidence base to inform interventions to improve health system responsiveness and equity. The subsequent sections outline the methodology, followed by the presentation of results, after which the findings have been discussed in relation to the existing literature, and the article concludes by highlighting key implications.

## 2 Methodology

### 2.1 Data source and study setting

This study utilizes data from a cross-sectional survey “Syrian Refugee and Host Community Surveys (SRHCS), 2015–16”, conducted in Jordan, Lebanon, and KRI (36), three countries that have hosted large numbers of Syrian refugees since the onset of the Syrian conflict in 2011. In Jordan, which followed a policy of holding refugees in camps, Azraq and Za'atari Camps for Syrians were the ones housing the most, followed by areas such as Mafraq and Zaqra, which were near the camps. Some refugees had moved out of the camps due to obtaining residency or work. The Amman governorate also had a high prevalence of Syrian refugees (37, 38). On the other hand, Lebanon adopted a non-encampment policy, hence the refugees were staying within host communities and in informal settlements (24, 38). In contrast, in KRI, Syrians as well as Iraqi internally displaced persons (IDPs) were free to move in and out of their different camps (37).

### 2.2 Survey design and sampling strategy

The surveys were implemented by the World Bank in collaboration with national statistical agencies and humanitarian partners, with the aim of generating comparable information on the living conditions, livelihoods, and access to services among both displaced and host populations (22). The data for this analysis were obtained through a formal access request to the World Bank Microdata Library.

Due to the absence of updated and comprehensive national sample frames, especially for forcibly displaced populations, mixed sampling strategies were adopted across the three settings. This approach combined stratified, multi-stage cluster sampling with purposive elements, drawing on auxiliary data sources, such as UNHCR registration data, satellite imagery, and local administrative information to construct workable sampling frames. In Lebanon and KRI, stratified multi-stage cluster sampling with probability proportional to size was employed which allowed population representativeness, whereas in Jordan the sampling design incorporated purposive elements outside camp settings due to the lack of an updated and accessible sampling frame. Given the varying national contexts, sampling methodologies were adapted accordingly:

- Jordan: The sample focused on Azraq and Za’atari refugee camps and nearby host communities in Mafraq, Zarqa, and Amman governorates. Sampling in camps was probabilistic and representative, whereas outside the camps, purposive sampling was employed in areas with high Syrian refugee concentrations based on available census indicators. A fully representative sample could not be achieved because of the inability to access the updated national sampling frame based on the 2015 Population and Housing Census. Consequently, the study relied on representative samples from Azraq and Za'atari camps and purposive samples from surrounding governorates and Amman, limiting representativeness to those specific areas rather than the national refugee population (22).
- Lebanon: Conducting a representative survey of all the refugees and hosts across Lebanon presented considerable challenges. A four-stage stratified sampling approach was adopted due to the lack of a recent census or existing enumeration areas in Lebanon. To ensure a representative sample, Circonscriptions Foncières (CF) were stratified by the proportion of Syrian refugees and selected using probability proportional to size. Each selected CF was further segmented using satellite imagery and local knowledge to delineate manageable enumeration areas. A full listing operation identified refugee and host HHs, from which a systematic sample of 40 HHs (20 Syrian and 20 host) per segment was drawn (22).
- KRI: Sampling drew on enumeration areas from the 2012 Integrated Household Socioeconomic Survey to ensure a representative sample of refugees and hosts across KRI. These areas were stratified based on the prevalence of Syrian refugees and IDPs. In each Primary Sampling Unit, up to 18 HHs were surveyed, aiming for an equal representation of Syrian refugees, IDPs, and host community HHs (22).

As described by Krishnan et al. (22), the survey instrument was implemented in Jordan, Lebanon and KRI, with minor adaptations to reflect the specific structure of refugee living arrangements in each setting. Despite these limited modifications, the core structure and content of the questionnaire remained consistent, enabling comparability across the three countries (22). The survey instrument comprised a standardized set of questions covering demographics, employment, access to public services, health, migration history, and perceptions before and after forced displacement. At the HH level, the head of HH was the primary respondent.

### 2.3 Variables

#### 2.3.1 Exposure

The exposure variable was the country of displacement. HHs were classified according to whether their members were displaced from Syria and were residing in Jordan, Lebanon, or KRI. A HH was considered a refugee HH if all the members held refugee status.

#### 2.3.2 Outcome

The primary outcome variable was OOP healthcare expenditure. Participants were asked whether they or anyone in their HH visited any kind of healthcare centre within the last 12 months. If they had, they were then asked whether any expenses were paid OOP at the point of care. Based on their responses, the data were categorized into two groups: “yes” for those who reported any amount of OOP payment, and “no” for those who accessed healthcare without any cost to them at the point of care. These responses were self-reported by the head of HH. Since this measure captured whether any payment was required, rather than the absolute amount spent, given differences in healthcare pricing, and health system structures across the study settings, the use of a dichotomous indicator facilitated more consistent comparison across the countries. Similar measures of out-of-pocket payment have been used in household survey-based studies to assess financial access to healthcare (17, 39).

#### 2.3.3 Covariates

The following covariates have been selected, which capture key sociodemographic characteristics of the head of HH, and HH size in order to account for potential influences on the outcome. The sociodemographic variables included were gender, marital status, highest level of education attained, employment status and age of the head of the HH at the time of the survey. Gender of the head of HH was recorded as binary variable, male or female. Marital status was categorized into single (which included never married, under-aged, widowed, separated, or divorced individuals) and married. For educational attainment, the highest completed level by the head of HH was classified into three groups: no education, primary level and secondary/tertiary level education. Employment status of the head of HH was divided into employed and unemployed. The age of the head of HH was a continuous variable. HH size was categorized into two groups: small HHs (1–5 members) and large HHs (>5 members). These covariates were selected based on existing literature and their relevance to both healthcare access and the ability to bear healthcare costs (13, 40)**.**

### 2.4 Statistical analyses

Descriptive statistics were used to summarise the characteristics of the study population, presented as frequencies, percentages and means with standard deviation (SD). To assess the association between displaced country and OOP healthcare expenditure, Generalized Estimating Equations (GEE) were used while accounting for the complex sampling structure of the data, assuming an exchangeable working correlation structure. Governorate or segment was used as the clustering level depending on the country-specific survey structure, allowing standard errors to be adjusted for correlation among observations within the same sampling unit. All models were adjusted for sampling weights provided within the dataset, except for Jordan. Jordan did not have sampling weight because it was not representative of the study population (22). Cases with no recorded responses were treated as missing at random (MAR). The extent of missingness was assessed for each of the variables and was found to be very limited (<5%), and therefore unlikely to affect the estimates (41). Odds ratios (OR) with 95% confidence intervals (CI) were calculated. In all regression analyses, Jordan was used as the reference category for country comparisons, due to its relatively more developed healthcare infrastructure compared to Lebanon and KRI (18, 19). Moreover, Jordan also exhibited the lowest observed prevalence of OOP payments in study sample, making it a suitable baseline for comparison when examining relative differences across countries. Statistical significance was defined as *p* < 0.05. All statistical analyses were conducted using IBM SPSS Statistics (version 28).

### 2.5 Ethical considerations

This study was based on secondary analysis of anonymised microdata obtained from the World Bank Microdata Library and the data collection was done in collaboration with other organizations including United Nations High Commissioner for Refugees (UNHCR). The datasets are publicly available for research purposes under the World Bank's terms of use (42), which require that all data remain confidential and be used solely for statistical and scientific research. All analyses were conducted at the aggregate level and no individual data was available to the authors. UNHCR has formal host-government authorization to collect and manage refugee data in all three settings: in Jordan, a 1998 Memorandum of Understanding (MoU), later amended in 2014 (43); in Lebanon, a 2003 MoU (44) complemented by a 2023 government–UNHCR data-sharing agreement (45); and in Iraq, MoUs with the Iraqi Ministry of Interior (2016) (46) and the Kurdistan Regional Government (2019) (47).

## 3 Results

### 3.1 Participants

**Figure 1:**
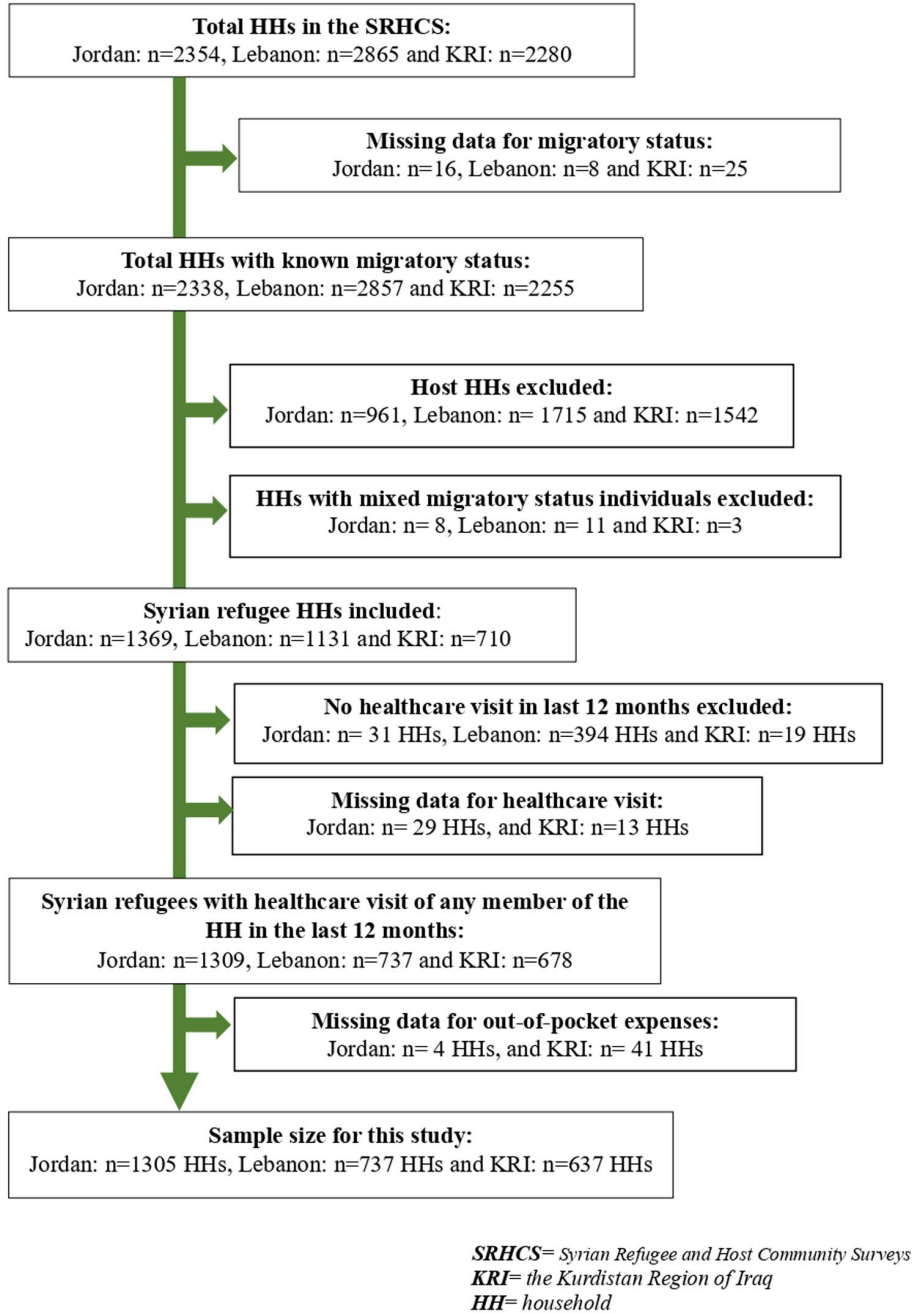
Flowchart of study population.

From the total HHs surveyed (2,354 HHs in Jordan, 2,865 HHs in Lebanon, and 2,280 HHs in KRI), HHs missing information on migratory status, host HHs and those HHs with mixed migratory status were excluded. HHs without a healthcare visit in the previous 12 months and those with missing data on OOP expenditure were also excluded. The final analytic sample included 1,305 HHs in Jordan, 737 HHs in Lebanon, and 637 HHs in KRI, each with at least one healthcare visit during the last 12 months and complete data on OOP expenditures (Figure 1).

### 3.2 Background characteristics of the study population

Table 1 summarizes the background characteristics of HH heads as well as HHs across the three host settings. The majority of HH heads were male across the three study settings and marital status patterns were largely similar across the countries, majority being married. Educational attainment varied, with a greater share of HH heads reporting no formal education in KRI (42.9%), compared to Jordan (29.6%) and Lebanon (25.0%). Majority of the HH head in Lebanon (80.0%) and KRI (64.3%) were employed compared to HH heads in Jordan (37.9%). HH size distributions and the mean age of HH heads were similar across the three settings. In terms of OOP expenditure, notable cross-country variations were observed, with lower proportion of refugee HHs in Jordan (39.5%) having OOP compared to 78.3% of HHs in Lebanon and 73.3% HHs in KRI incurring OOP expenditure for healthcare.

**Table 1:** Background characteristics of Syrian refugee households in Jordan, Lebanon and KRI.

Characteristics	Categories	Jordan (*n* = 1,305)	Lebanon (*n* = 737)	KRI (*n* = 637)
		*N* %	*N* %	*N* %
Gender of head of HH	Male	1,086 (83.2%)	667 (92.5%)	578 (90.4%)
	Female	219 (16.8%)	70 (7.5%)	59 (9.6%)
Marital Status of head of HH	Single	166* (12.9%)	75 (9.8%)	52* (8.9%)
	Married	1,118* (87.1%)	662 (90.2%)	579* (91.1%)
Highest Educational Level of head of HH	No education	386 (29.6%)	156* (25.0%)	267 (42.9%)
	Primary education	751 (57.5%)	483* (64.2%)	285 (41.9%)
	Secondary and tertiary education	168 (12.9%)	97* (10.9%)	85 (15.2%)
Employment Status of head of HH	Employed	487* (37.9%)	586 (80.0%)	422* (64.3%)
	Unemployed	797* (62.1%)	151 (20.0%)	209* (35.7%)
Age of head of HH (Mean ± SD)		40.5 ± 12.5	39.6 ± 10.8	42.3 ± 12.6
Household size	Small	656 (50.3%)	448 (49.0%)	405 (53.4%)
	Large	649 (49.7%)	289 (51.0%)	232 (46.6%)
Out-of-Pocket payment	Yes	516 (39.5%)	577 (78.3%)	412 (73.3%)
	No	789 (60.5%)	160 (21.7%)	225 (26.7%)

KRI, the Kurdistan Region of Iraq.

* The total sample size varied due to missing values.

The percentages for Lebanon and KRI are weighted.

### 3.3 Difference in OOP expenditure for households

The crude (COR) and adjusted odds ratios (AOR) for OOP health expenditure of refugee HHs in Lebanon and KRI compared to Jordan (along with the covariates) is presented in Table 2. The analyses were adjusted for key sociodemographic characteristics of the head of HH, and also HH size. Although some variations were observed across the covariates, the overall pattern of cross-country differences in OOP payments remained unchanged after adjustment.

**Table 2:** Crude and adjusted odds ratio for out-of-pocket expenditure of Syrian refugees including covariates in Jordan, Lebanon and KRI.

Characteristics	Categories	Crude		Adjusted^a^	
		OR (95% CI)	*p*-value	OR (95% CI)	*p*-value
Country of displacement	Jordan	Ref		Ref	
	Lebanon	5.54 (1.72, 17.8)	0.004*	5.25 (1.57, 17.5)	.007*
	KRI	2.78 (1.30, 13.6)	0.016*	4.50 (1.34, 15.1)	.015*
Gender	Male	Ref		Ref	
	Female	1.06 (0.58, 1.94)	0.86	2.62 (1.04, 6.62)	0.041*
Marital Status	Single	Ref		Ref	
	Married	1.35 (0.75, 2.41)	0.31	2.15 (0.98, 4.71)	0.057
Education	No education	Ref		Ref	
	Primary education	1.57 (0.98, 2.50)	0.058	1.56 (0.92, 2.64)	0.098
	Secondary and tertiary education	1.76 (0.92, 3.38)	0.089	1.92 (0.92, 4.03)	0.083
Employment status	Employed	Ref		Ref	
	Unemployed	0.70 (0.46, 1.06)	0.094	0.70 (0.41, 1.17)	0.17
Age		0.99 (0.98, 1.00)	0.35	1.00 (0.98, 1.02)	0.86
Household size	Small	Ref		Ref	
	Large	0.96 (0.62, 1.50)	0.87	1.00 (0.62, 1.62)	0.99

OR, odds ratio; CI, confidence intervals; KRI, Kurdistan region of Iraq.

* Shows statistical significance at *p*-value less than 0.05.

a Adjusted for gender, marital status, highest education, employment status and age of head of HH, and HH size.

The analysis revealed that the country of displacement was a strong predictor of OOP healthcare expenditure. After adjusting for other factors, it was seen that refugee HHs in Lebanon had more than five times higher odds of reporting OOP payments compared to those in Jordan (AOR = 5.25, 95% CI: 1.57, 17.5; *p* = 0.007). Similarly, HHs in KRI had significantly elevated odds of OOP expenditure, being over four times more likely than those in Jordan to report such payments (AOR = 4.50, 95% CI: 1.34, 15.1; *p* = 0.015).

## 4 Discussion

This study demonstrates significant variation in OOP health expenditures among Syrian refugees living in Jordan, Lebanon, and the KRI, i.e., the likelihood of incurring OOP payments differed significantly across these three contexts. Refugees in Lebanon reported the highest odds of OOP expenditure, followed by KRI, compared to refugees in Jordan.

A key contrast lies between Jordan's and Lebanon's health system model. Jordan had an explicit policy to house refugees in camps (22). Jordan's health system is characterized by centralized coordination between the national health system and humanitarian agencies (48). Refugees in camps such as Zaatari had access to free or subsidized primary care delivered by NGOs, while referrals to secondary care were partially subsidized (49). The availability of such subsidies may have resulted in relatively lower proportion of refugees in Jordan with OOP expenditure. In contrast to Jordan's approach, in Lebanon, refugees live among the host population and access the same highly privatized healthcare system that depends heavily on OOP payments. Refugees are excluded from national insurance schemes and rely largely on a mix of private providers and fragmented humanitarian assistance (27, 50). UNHCR subsidized some secondary care for the refugees in Lebanon, but assistance had been restricted to the most vulnerable and required co-payments by the refugees, leaving significant gaps in coverage (51, 52). This could explain the high OOP expenditure for Syrian refugees living in Lebanon.

KRI, on the other hand, showed an intermediate position between these two countries. The refugees in KRI lived both in camps and within the host community, with a very porous camp boundary that allows its residents to move freely and travel outside the camp (22). Camp-based health services offer free or subsidized care and basic healthcare access; however, limited diagnostic capacity, frequent medicine shortages, and weak referral mechanisms often compel refugees to seek costly private care outside the camp (53). A study in the camps in KRI showed that the majority of the participants went to private clinics or public hospitals outside the camp, which incurred eleven times and three times higher OOP cost, respectively, than camp primary health care clinics (30). In KRI, where camp-based services exist, gaps in service quality and availability may push refugees toward private care, thereby increasing OOP exposure. Such utilization of external facilities can contribute to financial strain for the refugees (77). Research on refugee camps in Turkey demonstrated that perceived service quality significantly influenced healthcare utilization and satisfaction among refugees, suggesting that limitations in quality, responsiveness, and trust in services may indirectly contribute to increased reliance on external, and often more costly, providers (54). Although both Jordan and the KRI adopted a camp-based strategy, the difference in OOP expenditure between the two settings indicates that such approaches alone do not determine financial burden. Research specifically focusing on KRI remains limited, but Iraq's broader health system context offers valuable insights as KRI and Iraq operate under the same national constitution (55). Iraq's comparatively weaker healthcare infrastructure has been linked to high OOP expenditures for vulnerable populations (56). Ensuring adequate resources, effective coordination, and overall strength of the national health system may contribute in preventing refugees from facing high healthcare expenses (8). Building on this, previous studies across refugee-hosting settings have also shown that limitations in system capacity, resource allocation, and policy design can significantly restrict access to affordable care, even where humanitarian support is present (57, 58).

Across the three contexts, the findings on refugees' OOP health expenditure reflect how each host country's healthcare system structure can shape financial vulnerability for refugees. Data from the Global Health Expenditure database indicate that in 2015, around the time of this study, OOP expenditure as a share of total health expenditure was lowest in Jordan, followed by Iraq and Lebanon (58). More recent evidences further show that OOP spending has remained high across Lebanon and Iraq. In Lebanon, for instance, OOP expenditure reached approximately 90% of total health expenditure in 2022, around 75% higher than the WHO-recommended threshold for CHE (59). Comparable vulnerabilities have also been documented in Iraq, where recent evidence indicates that a substantial proportion of general HHs continue to face CHE (40). Although these evidences do not directly capture refugee-specific spending patterns, it provided important contextual insight into the broader health system environments within which refugees seek healthcare. When considered alongside our findings, these contextual differences help to situate the observed variation in the likelihood of OOP payments across settings. Furthermore, while overall household health spending levels have historically been higher in Lebanon and Iraq than in Jordan, the continued reliance on OOP payments across all three contexts suggests that the financial burden associated with healthcare remains substantial for many households, with potential implications for refugee households as well.

These parallels indicate that the financial strain refugees experience cannot be viewed in isolation, rather it reflects systemic weaknesses in public health financing that affect all residents, citizens and non-citizens alike, reaffirming that refugee and migrant health is an integral part of public health (60). Efforts to improve refugee healthcare will remain constrained if host-country health systems themselves are underfunded, inequitable, or over-reliant on private expenditure (61, 62). When citizens face barriers such as high OOP costs, limited insurance coverage, and inequitable access, refugees, who are often in a more vulnerable state inevitably experience these challenges more acutely (63). Moreover, the challenges these refugees face may not only be due to the refugee crises but rather extensions of pre-existing structural and fiscal crises (64, 65). The refugee influx may have magnified these existing crises rather than causing them. Additionally, targeting refugee health services alone, for example through subsidies, without addressing similar challenges faced by host population, could impact social cohesion, especially in protracted displacement contexts (8, 66). Strengthening public health systems, therefore, benefits both populations i.e., it can reduce pressure on humanitarian aid, enhance equity, and build a foundation for sustainable, inclusive care (65).

Recent comparative analyses in other countries have also shown that while some countries integrate refugees (or other migrants) into their national health systems, others maintain parallel or restricted service models, resulting in uneven coverage and disparities in financial protection (67–69). These findings also show that refugees' healthcare experiences are shaped not only by their displacement status but also by the policy and institutional contexts of the host countries in which they reside. The differences in OOP expenditures across Lebanon, Jordan, and KRI reflect deeper inequalities in health financing. Protecting refugees from financial hardship may require not only targeted humanitarian assistance but also systemic reforms to strengthen national health systems, advancing health of refugees as well as hosts.

### 4.1 Strengths and limitations

This study draws on a large, multi-country dataset collected using a standardized questionnaire and harmonized methodology across three countries, Jordan, Lebanon, and the KRI. This consistency in data collection enhanced the comparability of findings and reduced variability due to methodological inconsistencies.

The survey tools captured socioeconomic and health-related information, enabling analysis of OOP expenditures among displaced populations. Although the data is not recent, similar patterns in healthcare barriers and financial hardship continue to be reported in recent studies among Syrian refugees such as in Jordan and Lebanon (70, 71). This indicates that our findings remain relevant and suggest that the situation has not improved, despite the record number of people who are forcibly displaced globally (2). This is even more concerning given that humanitarian aid has substantially and systematically decreased in recent years (72). These trends underscore the urgent need for more effective and sustainable interventions. As such, the findings reflect underlying system-level factors shaping exposure to OOP payments rather than time-specific conditions. Importantly, the value of this study lies not in identifying emerging patterns in healthcare financing, rather in providing a comparative assessment of refugee exposure to OOP payments across host countries with different health system structures as well as levels of humanitarian support. A key strength of this analysis is its explicit cross-country perspective, which allows examination of how the likelihood of incurring OOP healthcare payments varies across different host-country settings, rather than focusing on a single national context. Moreover, this time frame captures a critical phase of transition from emergency humanitarian response to structured healthcare for Syrian refugees in Jordan, Lebanon, and the KRI which occurred primarily between late 2014 and 2016 (73, 74).

Healthcare expenditure can vary substantially across contexts, and these differences can influence the OOP payments and factors such as the level of public sector involvement, financing mechanisms, and NGO support can influence the extent of OOP payments. Additionally, OOP costs can be influenced by the type and severity of illness or condition for which care was sought (17). The dataset did not include detailed diagnostic information that would allow us to distinguish between minor illnesses, chronic conditions, or emergencies. This can limit ability to assess whether higher reported costs reflect more severe health issues, frequent care-seeking, or differences in service pricing. However, in this study we did not measure the amount spent, instead, our analysis focused on whether refugee HHs reported any OOP payments. This provides useful insights into the extent to which healthcare is accessible without direct cost to refugees at the point of care across different host-country contexts. This study also did not assess the quality of care received, so lower reported OOP payments should not be interpreted as an indicator of adequate or high-quality healthcare. The survey captured OOP healthcare expenditures reported over the preceding 12 months, which provides a snapshot of healthcare-related financial experiences at that point of time but may not fully capture longer-term or cumulative financial risk exposure. Although the survey relied on responses from the head of the HH as they are usually familiar with income and healthcare matters, the use of self-reported data may introduce recall or even response bias, particularly for questions regarding healthcare expenditures. Additionally, the measure reflected the reported occurrence of OOP payments rather than independently verified transactions. As this study employed a cross-sectional design, causal relationships between variables cannot be established.

In this study, survey weights were available and applied for Lebanon and KRI to adjust for sampling design and representativeness. For Jordan, no weights were available, and all analyses were conducted using unweighted data. A representative national sample was not possible in Jordan because of the inaccessibility to the updated sampling frame from the 2015 Population and Housing Census (22). Instead, representative samples were drawn from Azraq and Za’atari camps and purposive samples from nearby governorates and Amman, meaning the findings are representative to specific areas only rather than the entire refugee population of Jordan. Overall, while the study aimed to generate comparable insights across contexts, differences in sampling frames, data availability, and implementation constraints resulted in variation in representativeness across countries, and therefore should be considered when interpreting the comparability of the country results.

## 5 Conclusion

This study highlights important differences in OOP health expenditures among Syrian refugees across Jordan, Lebanon, and the Kurdistan Region of Iraq. Our results demonstrates that variations in OOP experienced by refugees vary depending on the national context. Our discussion indicates that these differences may often be an extension of how public health systems are organised in host countries. Differences in OOP expenditures across settings may indicate that financial protection can be influenced by broader structural, policy, and health system factors. Strengthening these systems to reduce financial barriers and ensure equitable access is therefore essential not only for improving refugee health outcomes but also for advancing overall public health goals and system equity (75–77).

## Data Availability

Publicly available datasets were analyzed in this study. This data can be found here: https://microdata.worldbank.org.
